# Strategies for person-centredness at meeting places arranging group exercises for community-dwelling older persons– A focus group study from a stakeholder perspective

**DOI:** 10.1186/s12889-025-21843-z

**Published:** 2025-03-13

**Authors:** Daniella Dinse, Ulrika Olsson Möller, Marie Nilsson, Staffan Karlsson, Maria Haak

**Affiliations:** 1 Faculty of Health Sciences, Department of Nursing and Health Sciences, Kristianstad University, Kristianstad, SE-291 88 Sweden; 2 School of Health and Welfare, Halmstad University, Halmstad, Sweden

**Keywords:** Centres for the aged, Community network, Health promotion, Municipal government, Person-centred approach, Physical activity, Preventive health services, Older adults, Senior centres, Stakeholder engagement

## Abstract

**Background:**

Health-promotive interventions targeting older persons are important for active and healthy ageing. Hence, physical group exercises for community-dwelling older persons are arranged by various stakeholders via meeting places within the municipalities. Increased knowledge of how group exercises via meeting places can be arranged to promote health for the older population is needed. Therefore, the aim was to explore involved stakeholders’ experiences of group exercises for older persons arranged via meeting places in municipalities in relation to health promotion.

**Methods:**

Six focus group interviews were conducted online with 25 stakeholders from seven municipalities in Sweden. The stakeholders were managers of prevention units, municipal coordinators for physical activity, group exercise leaders, stakeholders from study associations, sports associations and private stakeholders, and non-profit stakeholders who arranged group exercises via the meeting places. The data was analysed using focus group methodology, where the focus of the analysis was to obtain the stakeholders’ collective understanding of the topic.

**Findings:**

The analysis resulted in two main themes and seven categories. In the main theme *Strategies to strengthen empowerment and exercise habits among older persons*, the stakeholders highlighted strategies on an individual level. Strategies of importance to attract new participants and supporting them in maintaining their exercise habits, empowering them through social belonging, adapting to older persons through responsiveness and evaluation, facilitating participation in decision-making, and enabling older persons to lead group exercises. The other main theme, *Strategies to strengthen the arrangement of group exercises over time*, highlighted strategies on an organisational level concerning financial resources, supportive environments, the importance of human resources, competence development as well as collaboration.

**Conclusions:**

Person-centredness emerges in health-promoting strategies both at the individual and organisational level. The study contributes to an understanding of how person-centredness is significant when working with health-promotive interventions for healthy ageing. Thus, a potential implication is to use a person-centred approach in the encounter with older persons and in the organisation when arranging group exercises for community-dwelling older persons.

**Supplementary Information:**

The online version contains supplementary material available at 10.1186/s12889-025-21843-z.

## Background

The need for health-promoting interventions targeting older persons has gained increased interest during recent decades. Health-promoting interventions have been highlighted by the World Health Organization [WHO] [1] as making an important contribution to active and healthy ageing. Globally, active and healthy ageing is important as more active people lead to a healthier world. International goals have been set as guidance for health promotion work at national level to strengthen all people’s access to supportive environments and to various opportunities to be physically active in daily life [1]. In Sweden, authorities follow these international goals to create an active society, environments, people, and systems [2]. One way to strengthen the environment and the people is through health-promoting interventions arranged for community-dwelling older persons over 65 years of age.

An important aspect of health-promotive interventions is physical activities. Health promotion is a process that involves creating opportunities for both the population and the individual person to increase control over and improve their health [3, 4]. Physical activity is considered a key contributing factor to physical health and mental well-being in general [5, 6]. Physical activity means any bodily movement [7], whereas exercise means planned, structured, repetitive and purposeful physical activity to improve or maintain physical fitness [8, 9]. Physical activity and exercise provide health benefits, regardless of age and functional ability [10, 11] and for older persons can, for example, reduce the risk of developing age-related diseases [12], depression [13, 14] and the risk of falls [15, 16]. Moreover, some choose to exercise in groups, which has been shown to be beneficial for older persons. The social support when older persons exercise in a group is valuable and may make it easier to continue exercising [17]. Long-term group exercises have been shown to improve muscle strength, and delay age-related declines in walking speed and physical function in older persons [18]. Furthermore, exercise, preferably in groups, can prevent or delay frailty in older persons [19].

For exercise to have a positive effect on health it is recommended for older persons to exercise with moderate intensity for at least 150 min a week [1]. In Sweden in 2022, 61% of men and 56% of women aged 65–84 self-reported that they met the recommendations [20]. Although many older persons achieve the recommended level, there is still a large proportion who do not. This emphasises the importance of health-promotive interventions that have the overall goal of supporting a person or group in taking power and control over their own circumstances, situations and goals; so-called empowerment. The basis of empowerment is that each person has the power, resources and capacity to identify their own needs and to find strategies to deal with them [21]. Hence, empowerment can be conceptualized on an individual level, but also on a group and community level. Still, people are always situated in a context, and the concept of empowerment entails relationships and social forces that influence the individual and have the potential to promote a sense of control and feelings of power [22]. In addition, empowerment can be conceptualized as both a process and an outcome. Empowerment is also an important concept within the theoretical framework of person-centredness [23], which is also a relevant approach within this field given that the older population is a heterogeneous group in terms of characteristics, experiences and conditions [24], which means that ageing is highly individual. In health promotion, as in a person-centred approach, it is important to start from the needs and wishes of the individual. It is also important to work from a holistic perspective and to enable participation where the individual can make decisions about their health and well-being [25]. Hence, it is important that older persons are as physically active as possible in accordance with their conditions [12]. Therefore, it is essential that municipalities enable all older persons to have the opportunity to exercise physically.

In Swedish municipalities, meeting places, also known community centres and senior centres, represent this kind of supportive environment that aims to create opportunities and support for older persons to improve their health by offering health-promoting interventions. Such health-promoting interventions are intended to strengthen the social, physical, and mental well-being and health [26, 27]. Via these meeting places in the municipality, group exercises for community-dwelling persons over 65 are arranged by various stakeholders, such as municipal stakeholders, private stakeholders, other community organisations and non-profit associations. Stakeholders can act both independently and in collaboration with each other. The benefits of stakeholder collaboration is that it can enable a diverse range group exercises to be arranged [26].

Various activities arranged via meeting places or similar venues have been examined from different perspectives. From the older persons’ perspective, social activities have been investigated by Hajak et al. [28] who found that the use of community centres in Germany was positively associated with life satisfaction among older male adults. Marquet et al. [29] demonstrated a positive effect on the level of physical activity in women who visited senior centres for exercise. Similarly, Hand et al. [30] demonstrated that inactive older persons who started exercising at senior centres improved their physical health and their health-related quality of life. Hickerson et al. [31] explored the older persons’ perspective of senior centres’ role in promoting physical activities in the USA. The study demonstrated that the organisational resources of the senior centre, the older person’s motivation and the social context contributed to enjoyable physical activity for the older persons [31]. In Sweden, Nilsson et al. [27] explored, from the stakeholders’ perspective, social meeting places for older persons, with a focus on promoting social fellowship, physical activity and good eating habits. The findings showed that there was a diversity of activities and meeting places for older persons, where both the public and non-profit sectors were responsible for the meeting places [27]. Another Swedish study from a stakeholder perspective [32], exploring group exercises arranged via meeting places during the COVID-19 pandemic, demonstrated challenges regarding communication and decision-making, the importance of adapting and finding new ways to offer group exercises, and highlighted the older persons’ ability to act for their own well-being. However, comprehensive studies solely focusing on physical activities via the meeting places from a stakeholder perspective are lacking.

Moreover, as health-promoting interventions arranged via the meeting places are part of the structure to promote physical activity in the municipalities, this is an important area to explore. Furthermore, stakeholder involvement is a key strategy in the Ottawa Charter [3] for successful health promotion. The area is also important to explore given the prevailing inequality that has been demonstrated between Swedish municipalities when it comes to creating conditions for health. Since there is a lack of a national structure in Sweden to promote physical activity, it differs between municipalities in terms of knowledge, resources and opportunities to create equal conditions for health [2]. In Sweden, only three per cent of healthcare expenditure is allocated to preventive interventions and therefore it is particularly important to explore whether existing interventions are functioning well [33]. Accordingly, the WHO [34] emphasised achieving the international goals to promote physical activity, and the importance of supporting collaboration and partnerships, strengthening professions and engaging in cross-sectoral collaboration. Such areas are relevant to the work of stakeholders when arranging group exercises via meeting places. As the stakeholders know about designing, collaborating, and arranging the group exercises via the meeting places, it is important to explore their perspective. Therefore, the aim was to explore involved stakeholders’ experiences of group exercises for older persons arranged via meeting places in municipalities in relation to health promotion. The study aimed to answer the following research questions:

- What do the stakeholders do in order for the older persons to benefit from the group exercises?
- In what ways do the stakeholders work in order to develop the group exercises?

## Methods

### Design and setting

A qualitative study with an inductive approach was conducted and analysed with the focus group method according to Krueger and Casey [35]. The stakeholders in this study had different roles and came from different organisations, but their key common ground was that they were all involved in arranging group exercise via the meeting places for community-dwelling older persons. Given this common ground, it was appropriate to use the focus group method, as it aims to develop a shared understanding of the research topic [36].

The study was part of a project in which several research questions concerned group exercises arranged via meeting places in municipalities [32]. The study was conducted in southern Sweden in municipalities with meeting places targeting community-dwelling persons aged 65 years and older. Meeting places were part of the municipality’s prevention unit and their use, by various stakeholders, was intended to strengthen the health of community-dwelling older persons. Meeting places could be in dedicated premises, online or outdoors. The function of the meeting places was to be local gathering spots, geographically dispersed within the municipality, where people could socialise and participate in various social, cultural and physical activities. These activities took place regularly and were often offered free of charge or at a low cost. However, only meeting places arranging group exercises were included in this study.

The meeting places were coordinated by municipal stakeholders and/or by other stakeholders such as non-profit organisations. Meeting places coordinated by municipal stakeholders usually had employed staff, unlike non-profit organisations coordinating a meeting place that used volunteer staff. Although one stakeholder was responsible for the coordination of the meeting places, different stakeholders could be invited to arrange group exercises via the meeting places. The stakeholders found in this study’s municipalities were municipal stakeholders, private stakeholders, adult education associations, sports associations, pensioners’ associations, and individual volunteers.

The number of meeting places, the stakeholder primarily responsible for coordination of the meeting place, number of employees, types of group exercises and cost of participation in group exercise activity varied between the municipalities (Table 1).

**Table 1:** Characteristics of the municipalities

Characteristics	*n* = 7
Urban (n)	2
Rural (n)	5
Meeting places (n) arranging groupexercises within each municipality	
md (min-max)	2 (1–17)
Municipal employees (n) at the meeting places	
md (min-max)	2 (0–10)
Municipalities (n) offering the following via the meeting places:	
Walking/hiking	7
Exercise gymnastics	7
Balance exercise	4
Dance	4
Sports school	4
Qigong	4
Strength exercise	3
Water aerobics	2
Other group exercise activities^a^	4
Municipalities (n) that offer:	
All group exercise activities for free	3
Partly free and partly at a cost	4
All group exercise activities at a cost	0
Mainly responsible for arranging group exercises via meeting places within each municipality (n)	
The municipality	3
Both the municipality and associations	3
Associations	1

^a^For example ball games, cycling, Nordic walking, yoga

### Recruitment and participants

In total, stakeholders working municipally for the well-being of older persons were contacted in nine municipalities. Of these, seven municipalities arranged group exercises for community-dwelling persons. To be included in the study the stakeholders needed to have experience in arranging different group exercises via meeting places in the municipality. A combination of purposive sampling and snowball sampling was used. The first stakeholder contacted in the municipalities was either one already involved in the research project or was found via the municipality’s website or switchboard. Each stakeholder who was contacted was also asked about who else was connected to the group exercises via the meeting places in their municipality. However, not all stakeholders mentioned who had a similar role within the same municipality, for example coordinators or volunteers, were contacted, hence the purposive sampling.

Each of the seven municipalities was represented by one or more stakeholders. A total of 39 persons were contacted and 25 chose to participate. To facilitate the discussions in the focus groups, the stakeholders were placed in homogeneous focus groups [35]. See Table 2 for the homogenous group composition. Although stakeholders with the same role were placed in the same group, heterogeneity was sought by varying the focus groups in terms of gender, municipality affiliation and years of stakeholder experience. Furthermore, in some cases, the stakeholders had dual roles and decided for themselves which focus group they would participate in. Furthermore, this study took place within a larger data collection for two studies. For one study, the interview questions concerned the arrangement of group exercises during the COVID-19 pandemic that was ongoing during the data collection [32], and the other, i.e. the present study, explored the arrangement of group exercises pre-pandemic. Due to the larger data collection, two of the stakeholders in this study only had experience of arranging group exercises via the meeting places during the COVID-19 pandemic.

**Table 2:** Characteristics of focus group participants

Characteristics	*n* = 25
Age	
Mean (min-max)	54.4 (34–92)
Gender	
Women (%)	19 (76)
Men (%)	6 (24)
Stakeholders	
Group 1 a	
Decision-makers	2
Managerial support	1
Group 1 b	
Decision-makers	3
Managerial support	0
Group 2	
Coordinators	5
Group 3	
Group exercise leaders	5
Group 4	
Private stakeholders	1
Sports associations	2
Adult education associations	1
Group 5	
Pensioners’ associations	3
Individual volunteers	2
Experience in a stakeholder role in years, md (min-max)	
Group 1	5.7 (3–15)
Group 2	6.5 (1-13.5)
Group 3	6 (2.5–15)
Group 4	3.5 (2–11)
Group 5	6 (1–10)

Note. In group 1 a, there were dropouts and only a few stakeholders. To ensure variation in the material, another focus group 1 b was conducted

### Data collection

Between June and November 2021, six focus groups were conducted. At this time, the COVID-19 pandemic was ongoing with its restrictions. Therefore, the focus group interviews were conducted and recorded via an online video communication platform. All stakeholders were invited to test the online video communication platform before the focus group. To increase the quality and interaction within the focus group, all stakeholders were given instructions to always have both the camera and microphone on. All the stakeholders had the cameras on during the focus groups.

As recommended by Krueger and Casey [35], one moderator and one assistant moderator conducted the focus group interviews. The moderator initiated the focus group interviews by providing general information and explaining the aim of the study. Thereafter, the moderator informed about the voluntariness of participation and emphasised that the stakeholders could withdraw their participation at any stage without any negative consequences. Then everyone introduced themselves. After that, the moderator started the focus group interview with a general request: “Please tell us about the group exercise activities that you are involved in via meeting places in the municipality”. An interview guide asking stakeholders to share their pre-pandemic experiences was used in all focus group interviews. The interview guide was developed by the research team, drawing on established knowledge in the field and previous studies [26, 27] and health promotion concepts (see Additional file 1). The moderator led the discussion and encouraged the stakeholders to share their perspectives. Also, to facilitate an in-depth discussion and to obtain additional information, both the moderator and assistant moderator used prompting questions. The collective experiences in a focus group could, however, steer the direction of the discussion and thus the discussion could differ slightly between the focus groups, in accordance with Krueger and Casey [35]. At the end of the interview, the assistant moderator made a summary of the discussions. Furthermore, field notes were taken by the assistant moderator. The field notes contained themes and key points and described the interaction within the focus group. All stakeholders were involved in the discussion, and the interaction between them was good. The recorded interviews lasted between 83 and 104 min but due to the ongoing COVID-19 pandemic the discussions also contained questions about arranging group exercises during the pandemic. These aspects are highlighted elsewhere [32]. A reflection took place after each focus group where the moderator and the assistant moderator reflected on their experiences of the session and wrote notes.

Four of the focus groups were led by DD and UOM as moderator and assistant moderator. In two of the focus groups, MN and MH replaced DD and UOM since they were known to two of the stakeholders.

Focus group interviews were recorded audio-visually and transcribed verbatim. The first by DD and the rest by a professional transcriber. All transcripts were validated by DD by comparing the transcribed text with the recordings. The stakeholders were de-identified in the transcribed text and all data were handled confidentially. In the case of the two stakeholders, who had experience in arranging group exercises during the COVID-19 pandemic but not before, the transcripts of the focus groups in which they participated have been reviewed by the DD. The review showed that they did not add anything additional or new compared to the other stakeholders and therefore it was not considered relevant to exclude them from the focus groups. Their participation was not considered to affect the data. No quotes with content from them have been used.

### Data analysis

The analysis was based on a stepwise procedure as described by Krueger [37, 38]. The focus of the analysis according to the focus group method is the group perspective and not the individual perspective. The discussions taking place in the focus group form the basis for a collective understanding of the research topic [35]. As support in the interpretation process, notes made during and after the focus groups were used.

The analysis was carried out by taking the following steps. First, the recordings were reviewed repeatedly by DD and UOM and the transcripts were read several times to gain an understanding of the data. Second, each focus group interview was reviewed separately. DD and UOM individually identified repeated themes, concepts and patterns throughout the material from the six focus groups. Their findings were then compared with each other. From here, the analysis was performed jointly by DD and UOM. Next, preliminary themes were identified that were relevant to the aim of the study. Here, attention was paid to topics that were repeated frequently, themes that seemed central to participants, and differences in perspectives and opinions between participants. Relevant quotes and expressions that matched the different themes were highlighted. Then similar quotes were grouped together to create categories. After this, DD sorted the emerging themes and underlying categories using the NVivo 2022 software. To ensure that the themes and underlying categories matched the meaning of the data, they were continuously revised. Third, to get an overview of the material, the data was condensed under each category and abstracted to some extent by DD. Then, short descriptive summaries of each category and theme were written to serve as a basis for the next step of the analysis. In the descriptive summaries it was important to bring out the participants’ collective view on the topic. Fourth, this summary was used as the basis for interpreting the material [37, 38]. The interpretation aims to capture and understand the stakeholders’ collective understanding of the subject. In this last step, all authors critically revised the emerging findings to reach consensus and a shared understanding of the focus group interviews.

All stakeholders were invited to a member check [39] and nine participated. Those who participated represented four focus groups and five municipalities. The member check was carried out on two occasions online and the preliminary findings were given through an oral presentation. The findings were discussed, and the discussion allowed the stakeholders to add anything they omitted to share at the previous focus group sessions, but no new information came up. Instead, the stakeholders reflected and confirmed the findings, and the researchers’ understanding was deepened after the member check.

### Ethical considerations

When conducting audio-visual focus groups there are ethical aspects to consider. For instance, audiovisual focus groups can make participants feel an increased sense of anonymity and more willing to share their opinions [40]. To increase confidentiality, all stakeholders were given instructions not to pass on anything said during the focus group. However, the stakeholders were informed that they were welcome to contact each other afterwards for further exchange between each other. Furthermore, in audio-visual focus groups, the participants themselves decide where to sit during the interview [41]. Therefore, before the focus groups, the stakeholders were informed in both written and oral form about important ethical aspects regarding online audio-visual focus groups. For example, stakeholders were instructed to sit in a quiet environment to avoid being distracted. If there was a risk that someone outside might hear what was said during the focus groups, the stakeholders were asked to use headsets. Also, the stakeholders were informed about the possibility of using virtual backgrounds if they wanted to protect their privacy from public view. However, the research topic was not considered to be of a sensitive nature and therefore the risk of harm was considered small.

## Findings

**Fig. 1:**
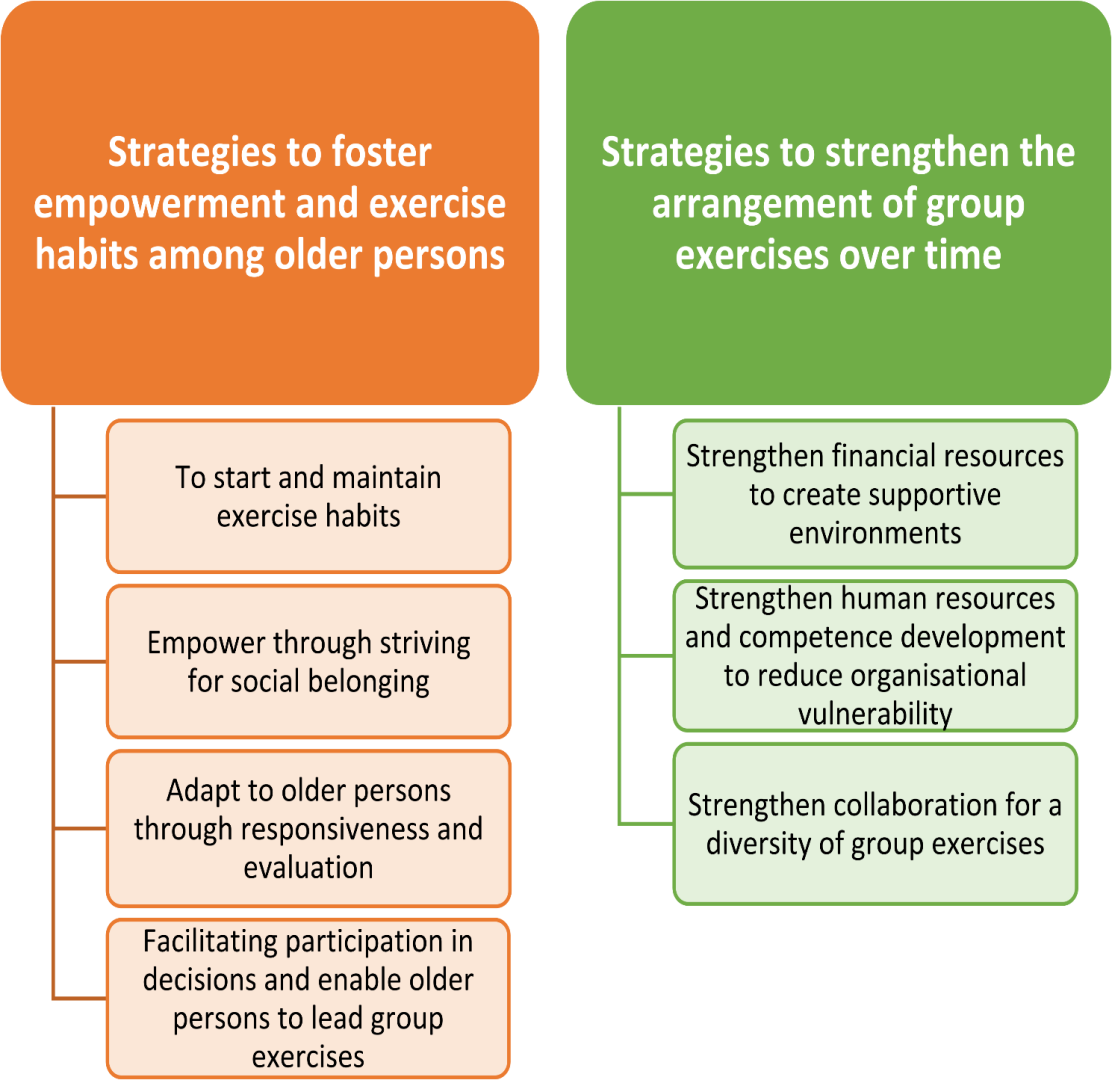
Themes and categories

Stakeholders’ experiences of arranging group exercises are presented in two themes and seven categories illustrating stakeholders’ different strategies at an individual level in the theme *‘Strategies to foster empowerment and exercise habits among older persons’* and at an organisational level in the theme *‘Strategies to strengthen the arrangement of group exercises over time’* (Fig. 1).

### Strategies to foster empowerment and exercise habits among older persons

Stakeholders’ strategies to foster empowerment and exercise habits by strengthening the older persons as persons are illustrated in the categories *To start and maintain exercise habits*,* Empower through striving for social belonging*, *Adapt to older persons through responsiveness and evaluation* and *Facilitating participation in decisions and enable older persons to lead group exercises.* However, the strategies did not come without challenges, which was also expressed in the categories.

#### To start and maintain exercise habits

Attracting new participants to start and to continue exercise in groups was considered an important strategy to strengthen older persons’ exercise habits. Men, inactive older persons and persons with a foreign background were considered as groups who, due to individual prerequisites, needed particular support to start exercising. The stakeholders demonstrated different methods to attract the older persons to group exercises. All available forums were used to reach out, such as health centres, social media, and newspapers. The stakeholders agreed that the best way to attract was when the older persons themselves told others how they enjoyed the group exercises and felt improved health. These motivating older persons were described as acting like a catalyst and, through their positivity and by being active in group exercise, they attracted new participants.

Besides the ambition to attract new participants, the stakeholders also described strategies to strengthen and maintain exercise habits. One way to encourage continued exercise was to guide older persons to find new activities when needed. For example, the stakeholders set aside time at the end of the term to talk about possible future exercise activities. Stakeholders agreed that it was important for the older persons to continue in the same routine. Keeping the same time and structure for the activity over time was perceived as beneficial as it made it easier for the older persons to continue exercise and thereby strengthened their exercise habits.

#### Empower through striving for social belonging

The social belonging that came with the group exercises was something that the stakeholders perceived as empowering for the older persons. Friendly relationships were created, and a sense of social belonging emerged within the group. From the combination of social belonging and physical exercise, a strong group cohesion emerged. This was described as particularly beneficial and an important motivator to continue exercise habits in groups via the meeting places, as exemplified in the following quote:

*Interview person [Ip] 24: (…) I experience that they contact each other when someone is trying to slip away and then it’s the group that calls and says ‘Hey*,* we didn’t see you on Tuesday*,* how’s it going?’ and so on. We notice that they care a lot about each other and even if new participants appear*,* they are a core group that exists constantly.*

*Ip 25: Yes*,* but then*,* as you say [Ip 24]*,* the social part becomes one of the success factors.*

*Ip 24: Mm.*

*Ip 25: So*,* the activities build up the social part*,* strengthen the social context for those who participate and make them want to continue.*

*(Focus group no.1 b)*

A strategy highlighted by stakeholders in their effort to achieve social belonging was to see and notice each person, which made them feel welcome and included. The stakeholders described a need to be constantly present with all their senses and to notice signals from the older person as everyone was unique, with their different backgrounds and needs. The importance of being present in the encounters as a stakeholder and being sensitive to signals from the older persons is exemplified in the following quote:

Ip: 17 (…) they [stakeholders arranging group exercise] must be there present all the time with all their senses really, so more like friends with our participants, instead of staff and visitors… that’s how I experience it.

Ip 15: Mm.

Ip 13: Mm.

*(Focus group no. 2)*

#### Adapt to older persons through responsiveness and evaluation

To adapt according to the needs and wishes of older persons was considered an important strategy for striving towards empowerment. To be able to adapt, it was considered important to be responsive and to conduct evaluations of the group exercise activities and health improvements.

In terms of being responsive, i.e. being attentive and listening to the needs and wishes regarding implementation and development of group exercise activities it was considered important to encourage the older persons to share their wishes, either in person or via social media. To enable this the stakeholders described how they strived to make the older persons feel that both the stakeholder and they were members of the group on equal terms. Although the stakeholders wanted to be responsive to the older person’s wishes, it could be challenging to accommodate them due to insufficient prerequisites. In line with the ambition to be responsive and to adapt, the importance of adjusting the pace of the exercise, the level of difficulty, the group size or composition, and the use of modern and exciting music was highlighted. The stakeholders experienced that many activities had been adapted and improved through an exchange between the older persons and the stakeholders. By being responsive to and adapting to the older person’s needs and wishes the older persons were perceived as being empowered. How the group exercise activities were adapted by responsiveness is exemplified in the following quote:

*Ip1: (…) then they come up with requests… ‘Oh*,* couldn’t we do that’ and so on… and then we try to make it happen as far as possible*,* we can help and fix.*

*Ip2: You also get very sensitive ears*,* so you can hear what they’re discussing maybe over coffee or while walking together in pairs and you can even pick up what the people at the back are saying and thinking… you become very responsive.*

*Ip1: Yes*,* very responsive indeed.*

*(Focus group no. 3)*

In terms of adapting through evaluation, it was important to have evaluations of both the group exercise activities and health improvements in the older persons. Evaluations were considered to empower older persons by giving them the opportunity to have a say in group exercise activities and by providing them with insight into their health improvements. Furthermore, by evaluating group exercise activities and health improvements, it could be ensured that the activities were relevant and effective.

Evaluations of the group exercise activities were mainly carried out by asking the older persons for their opinions during or after the exercise session. The results were then used to adapt the activities to their needs and wishes. Evaluations were also performed by counting the number of participants. Having few participants indicated that the activity was not attractive, while many participants indicated that it was. However, this was considered misleading as it said nothing about the quality of the group exercises. However, evaluation instruments were considered lacking, and thus important to develop, as better instruments could lead to better adaptation to the older persons. Moreover, through evaluations, the stakeholders saw opportunities to raise the issue of the need to adapt, expand and develop group exercises with politicians, to make them better for older persons.

Another evaluation strategy was to evaluate older persons’ health improvements through a variety of methods, including subjective observations, questionnaires on self-rated health and quality of life, and professional evaluations of physical functions such as walking speed and balance. These evaluations were used to identify areas for improvement, to adapt exercise and to motivate continued exercise, all of which were considered to empower older persons to take control of their health and well-being. How older persons’ evaluations of health improvements were used in practice to adapt is shown in the quote:

*Ip 10: One asked a bit about sleep quality before starting the group and after the group and a bit about everyday things (…) and one also measured the physical age with a special scale and they found that quite funny (…).*

*Ip 9: (…) evaluation and measurement are important for both the municipality and the individual participants. Often*,* the participants themselves may not feel that there has been any difference*,* but then we have proof that there’s indeed a difference and that motivates them very much to continue. At the same time*,* if we notice that something is lagging behind*,* for example*,* if we specifically practised balance*,* but the balance has actually worsened*,* then we can tell those who arranged the exercise (…). And then*,* the municipality also gets answers about whether what they’re doing is effective and what kind of effect it has.*

*(Focus group no. 4)*

#### Facilitating participation in decisions and enable older persons to lead group exercises

Facilitating participation in decisions was considered an important strategy to foster empowerment. The older persons’ degree of participation in decision-making could vary and therefore stakeholders perceived that this needed to be increased. Stakeholders perceived that, in some cases, decision-makers in municipalities based their decisions on group exercise activities mainly on what exercise experts considered important instead of discussing with the older persons first. This was considered problematic as it was perceived that older persons wanted to be involved. Thus, to increase older persons‘ participation in decisions, it was recommended to somehow directly address them by, for example, setting up an older persons’ council and meeting with pensioners’ associations. The quote below illustrates this:

*Ip 23: Then we’re going to have something called an older persons’ council*,* where we want to get in touch (…) and discuss with older persons and especially find out how they think about their ageing and staying active (…) what do they want*,* what do they want at different ages (…) because older persons are not one group*,* they are different just like everyone else. And how do we meet that (…).*

*Ip 25: (…) we have very strong pensioners’ associations (…) and they come up with new things all the time*,* wishes*,* opinions*,* and are involved (…).*

*(Focus group no. 1 b)*

Furthermore, striving towards mutual responsibility for the meeting places and group exercise activities was a strategy designed to foster empowerment. Mutual responsibility could also be shown through encouragement to step in as leaders if the older person wanted to or if the stakeholder was unable to. The involvement of older persons as leaders is exemplified below:

*Ip 8: Often you find someone in the organisation who might come up with an idea… I think sometimes we think we are so good at knowing what to arrange at our meeting places*,* but we also need to allow our participants to come up with their own ideas… and if they come up with their own idea*,* we can say: ‘Yes*,* but then maybe you can be responsible for it’.*

*Ip 7: Yes.*

*(Focus group no. 1 a)*

#### Strategies to strengthen the arrangement of group exercises over time

The stakeholders experienced that it required strategies at an organisational level to maintain group exercise activities at the meeting places over time as illustrated in the categories *Strengthen financial resources to create supportive environments*,* Strengthen human resources and competence development to reduce organisational vulnerability* and *Strengthen collaboration for a diversity of group exercises*. However, strategies did not come without challenges, which is demonstrated in the categories.

#### Strengthen financial resources to create supportive environments

Stakeholders said that strengthening long-term financial resources was an important strategy to create supportive environments and thus maintain group exercises. Hence, it was considered important to work towards a strong dialogue between stakeholders and politicians. Supportive environments were discussed in terms of the location of the meeting places and their surrounding environments, for example whether they had access to green areas and outdoor gyms. Also, appealing, adaptable and physical accessible premises with sufficient indoor space were emphasised as important for the continuity of group exercises over time. However, not only physically environments were discussed. Offering group exercises as cheaply as possible was another strategy raised to increase accessibility, as illustrated in the following quote:

*Ip 18: (…) we have always endeavoured to have very low fees*,* so there’s no one who can’t afford our activities.*

*Ip 22: I don’t quite agree with you there*,* I know several people at the meeting places*,* where I volunteer who don’t have a single penny to spare.*

*Ip 18: Yes.*

*Ip 22: No*,* so I don’t think we can be entirely sure of that. There are many older women who live almost below the subsistence level*,* on their pension.*

*(Focus group no. 5)*

However, achieving long-term financial resources was emphasised as challenging. Politicians were regularly replaced due to elections, which was perceived as making long-term planning difficult. Stakeholders emphasised that it was difficult to show results of investments within a short period of time. It could happen that financial resources were withdrawn or reduced before outcomes were visible. Experiencing reduced financial contributions in favour of other groups of people is exemplified in the following quote:

*Ip 11: (…) but now the municipality reduced the contribution to the study associations so that they could invest in children and young people instead… but there are already a lot of activities for children and young people (…)*.

*Ip 12: Mm (…) we think that there must be a long-term plan*,* and we want [sports schools for seniors] to be included as part of the political programmes (…) that there should be something for this target group. Just like they offer sports schools for children*,* which they are very good at (…).*

*(Focus group no. 4)*

#### Strengthen human resources and competence development to reduce organisational vulnerability

Strategies discussed to strengthen the group exercises over time were from an organisational perspective, designed to strengthen competence development and the human resources associated with them. It was generally considered that there was a need for more municipal staff working with the group exercises, as low staffing increased vulnerability and reduced the number of activities that could be offered.

Strengthening competence development among stakeholders was considered an important strategy for arranging group exercises over time. It was common to discuss topics such as active and healthy ageing within the stakeholder group and it was experienced as important to use the acquired competences to discuss with the older persons in order to pass on the knowledge to them. Furthermore, competence development in the form of specific training programmes on active and healthy ageing was considered necessary as the stakeholders had different professional backgrounds and perspectives. However, such training was not common and therefore this area was considered important to improve, as shown in the following quote:

*Ip 6: (…) sometimes I see that if you come in as a nurse assistant and start working in a preventive unit*,* you’ve skills in nursing*,* you should take care of people and you should help*,* and you can see that this professional caring perspective differs from other professional backgrounds.*

*Ip 8: Mm.*

*Ip 6: So*,* as staff*,* you need competence development. It’s also very important to think about preventive activities (…) now we’re talking about healthy older persons*,* or seniors*,* we should engage in health promotion*,* for them to develop*,* to maintain*,* to strengthen their ability to continue living in ordinary housing*,* and to feel needed.*

*Ip 8: Mm.*

*(Focus group no. 1 a)*

Volunteers as stakeholders were experienced to be an important human resource to maintain the group exercises over time. Although most of the volunteers were older persons, getting them to become and remain volunteers was challenging and was emphasised to require great commitment from the ordinary stakeholders. Therefore, strategically building volunteer work and supporting volunteers was time-consuming, but necessary for them to remain volunteers. This is shown by the quote below:

*Ip 15: It‘s difficult*,* with volunteers*,* so*,* it‘s an art to keep it going year after year.*

*Ip 13: Mm (…) Managers and politicians think that just because volunteers are in charge of activities*,* staffing resources are not required. It takes lot of staffing resources because you must clap your hands and cheer a bit [for their effort] to make it work… sometimes… they can‘t function without a stimulus*,* the volunteers*,* and I feel that the stimulus is of course the exercise group participants’ gratitude and joy*,* but [providing] the stimulus also takes so much from me*,* from my resources.*

*(Focus group no. 2)*

Additionally, part of the strategy to strengthen human resources included constantly empowering each volunteer by recognising and praising their work. It was also important to officially recognise the volunteers, no matter how big or small their contribution was.

#### Strengthen collaboration for a diversity of group exercises

Strengthening collaboration was an important strategy to maintain the arrangement of the group exercises over time but also to enable a diversity of activities. With a strong collaboration between stakeholders, it was experienced that a greater diversity of group exercise activities could be achieved. Having more stakeholders collaborating in the arrangement of group exercises could also strengthen the possibilities to maintain group exercises over time. Factors facilitating collaboration were a clear division of responsibilities and a good transfer of information between them. If, on the other hand, there was limited or no collaboration between stakeholders, it was challenging, especially if all collaboration was dependent on one stakeholder. This was perceived as a vulnerability and resulted in fewer activities. Furthermore, in municipalities where associations arranged most of the group exercises themselves, collaboration with the municipal stakeholders was experienced as insufficient. Then it was desired that someone from the municipality had an overall responsibility for collaboration. Accordingly, collaboration was something that stakeholders asked for and wanted to strengthen.

## Discussion

This study aimed to explore involved stakeholders’ experiences of group exercises for older persons arranged via meeting places in municipalities in relation to health promotion. The findings were presented from two perspectives. Firstly, the stakeholders highlighted at an individual level health promotion strategies towards the older persons in terms of fostering empowerment and exercise habits among them. Secondly, stakeholders highlighted health promotion strategies at an organisational level in terms of strengthening the group exercise arrangement over time. Through these strategies highlighted by stakeholders in the context of arranging health-promoting group exercises, similarities to the person-centred approach also emerged, which is what will be discussed below based on the findings of this study’s results.

The stakeholders highlighted various strategies to attract older persons to start and maintain exercise habits. The strategies highlighted in our findings is in line with a person-centred approach [23]. That is being responsive to older persons prerequisites, needs and wishes. Such strategies have also been identified by other studies as important factors for older persons to start and to continue to exercise over time [17, 42]. Furthermore, other studies [43, 44] demonstrated that an important motivator for older persons to start and continue group exercises was having available staff who both encouraged and, through their expertise, provided safety for the older persons. This demonstrates the important role of stakeholders in strengthening the exercise habits of older persons but also consciously striving to foster empowerment at an individual level.

Our study shows that to foster exercise habits among older persons, it is important to work strategically to facilitate social belonging within the group exercise activities. The stakeholders experienced that feelings of social belonging were empowering and acted as an important motivator for continued exercise habits among the older persons. This is in line with research from Hickerson et al. [31] and Hosokawa et al. [45] where the social environment in which physical activities take place is important for community-dwelling older persons’ participation. Since the social environment contributes to good exercise habits and thus also to the promotion of health, it can be likened to a healthy environment. Similarly, the experience of being in an environment with a healthy culture, where the atmosphere is good and good relationships are created, is an outcome of a person-centred approach in the organisation [23]. This in turn shows the important role of person-centredness in health promotion interventions like group exercises. Given this, promoting physical activity and exercise habits among older persons involves much more than the explicit arrangement of group exercises activities. Our findings show that getting older persons to participate in group exercises over time requires awareness and strategic work. It is well known that social belonging is of importance for the practice of physical activity [46]. Furthermore, Komatsu et al. [47] show the importance of group exercise leaders work towards creating an environment where participants socially support each other and where they are encouraged to return regularly. Thus, our research suggests stakeholders to work strategically towards strengthening older persons’ sense of social belonging in group exercise activities.

The present study highlighted the importance of strategies for competence development within the area of active and healthy ageing. Due to the stakeholders’ varying perspectives and backgrounds a continuous opportunity for reflection and competence development is a prerequisite in a person-centred approach [23]. One can also assume it has the effect of promoting empowerment among stakeholders. Our study shows that competence development mainly took place through discussions between the stakeholders who in turn passed on their newfound knowledge to the older persons who participated in the group exercises via the meeting places. However, the stakeholders requested more specific competence development in active and healthy ageing in the form of formal training programmes. This is in line with international competency frameworks for the public health workforce which call for training in areas such as promoting social participation, empowering citizens, having the ability to assess health needs and evaluating health promotion interventions and programmes [48]. It is particularly important that front-line health promoters, i.e. those who meet the target group, receive training in health promotion to enable them to support older persons in maintaining their health [49]. The stakeholders are the people closest to the older persons and are thus the ones who can encourage and help the older persons to make positive changes in their health. When stakeholders have more knowledge about what is needed for group exercises to be health-promotive and what contributes to sustainable exercise habits among older persons, they have an opportunity to influence the organisation. Through increased competence, more targeted and more high-quality interventions can be created for older persons.

Our findings showed that it was crucial for stakeholders to work also on an organisational level with strategies for long-term financial support. A supportive organisational system with prerequisites for running an organisation over time is an important part of a person-centred approach [23]. The stakeholders experienced that financial support was short-term and changeable, varying depending on which political party was in power in the municipality. In line with Loss et al. [50] our findings show the importance of financial support from the local government for arranging group exercises over time. Furthermore, to receive financial support from the local government, the stakeholders in our study perceived that the group exercise’s positive effect on health and well-being needed to be demonstrated. Thus, person-centred strategies at individual level, for example evaluations, as illustrated in the category “Adapt to older persons by responsiveness and evaluations” can be useful at the organisational level to demonstrate group exercise activities effects on health. Research has shown cost-effectiveness as well as health effects. A study by Snowsill et al. [51] highlighted, by using health-related quality of life instruments, that one year of group exercising for older persons led to sustained improvements in quality of life and that the intervention was cost-effective. Other studies [52, 53] have also demonstrated that community-based group exercises are cost-effective. Consequently, that group exercises both increase the quality of life of older persons and are cost-effective can therefore be used as an incentive for local authorities to provide a long-term financial plan for group exercises arranged for older persons via meeting places. Prerequisites that are person-centred are needed to strengthen supportive organisational system over time. This in turn provides stability for the older persons as it enables them to continue exercising via the meeting places which in turn enables them to strengthen their exercise habits and thereby also promotes healthy ageing.

To sum up, person-centredness emerges in the health-promoting strategies presented by the stakeholders. This study brings an understanding of how person-centredness is significant when working with health-promotive interventions for healthy ageing. Given that it is the older persons who are the focus of the group exercise, it is important in future studies to explore their perspective on whether the group exercise activities via the meeting places promote health and are person-centred.

### Methodological considerations

Methodological considerations for this study will be discussed based on the trustworthiness concept of Lincoln and Guba [39]. In a focus group discussion, the participants can share thoughts, experiences, and perceptions. Therefore, this method suited the aim of this study well as all involved stakeholders had the same key common ground in regard to arranging group exercises via the meeting places. The fact that they all shared the same common ground but had different roles as stakeholders enabled more perspectives in the same field. Also, the discussions in the focus group can provide insight into a range of perceptions and experiences from stakeholders that would be less accessible in individual interviews [35]. Stakeholders were placed in homogeneous focus groups to reduce the risk of the power imbalance that could have arisen given the different roles that stakeholders had, e.g. manager and exercise leader, in arranging group exercises via the meeting places. While this can be seen as a strength, it would still have been interesting to conduct a focus group that included stakeholders with different roles to see if additional dimensions emerged. Furthermore, in order to have good discussions, it was important that the participants were persons who knew the field well and had varied experience of it. Through purposive and snowball sampling, each municipality was contacted to find everyone who was involved in the group exercises via the meeting places. This resulted in a selection of 25 stakeholders with varying backgrounds, regarding factors such as age and stakeholder role. Such variation in selection strengthened the study’s credibility. However, there were two stakeholders who had no experience of arranging group exercises via the meeting places before the pandemic. The data material has been reviewed to ensure that they did not influence the discussions (which they did not). No quotes from these stakeholders have been used. In this way, credibility was considered to have been ensured [39]. In order to share the stakeholders’ experiences, it was important to have an open and permissive climate during the focus group interviews. To enable such a climate and to create good opportunities for interaction, homogeneous focus groups were chosen [35, 38].

Furthermore, due to the ongoing COVID-19 pandemic, the focus group interviews were conducted online via a video conference system. Everyone who participated was able to handle this forum and all participants chose to have a camera on for good interaction. Regarding conducting the focus groups online, special considerations regarding privacy and confidentiality need to be made [41, 54]. In online focus groups, it is the participants themselves who decide where to sit during the meeting. All participants therefore received both oral and written information about factors to consider to maintain privacy and confidentiality prior to the focus groups. For example, they were informed that to protect their own privacy they could use a video background. They were also informed that they were not allowed to record the interview and that they should sit so that others outside the focus group could not hear the interview, which could be achieved, for example, by using headsets. However, the discussion topic of this study was not considered to be of a sensitive nature, which reduced the risk of harm. Additionally, through the online focus groups, the recruitment of stakeholders was facilitated as it was easy to participate and saved time on travel, factors which were considered strengths. Furthermore, the interaction in a focus group can be affected if there are too many or too few participants. The focus groups had three to five stakeholders. Usually, four to eight participants are recommended in a focus group where the participants meet physically [35]. However, in an online focus group it can be beneficial for the interaction to have smaller groups as the groups then become easier to manage [40, 41]. However, the experience of the focus groups was that all participants were involved in the discussion and that everyone could freely express their experiences, which strengthens the study’s credibility [39].

To achieve confirmability, the part of the analysis where the discussion content related to the aim was identified was carried out separately by the first and second authors. Furthermore, all authors were involved in continuously revising the findings to ensure that the stakeholders’ experiences, not the researchers’ perspective, were reflected in the findings [39, 55].

To enable a similar study to be repeated in a similar context and with similar participants using the same procedure, the study’ design, implementation, context and participants have been clearly described. This strengthens the dependability of the study but also enables transferability [39, 55]. The findings of this study could be transferred to meeting places in, for example, other countries where health-promoting interventions are arranged for older persons. Furthermore, the findings of this study could also give an indication of how health-promoting interventions can be arranged in other contexts, such as in healthcare or for other persons where special needs for health-promoting interventions are needed.

Finally, we summarise strengths and weaknesses of the study. One strength was the recruitment of participants with several different roles in arranging group exercise for community-dwelling older persons and that both the municipality and the non-profit sector were involved. This gave variety to the data and reflects how it is organised in the municipalities. Furthermore, the online focus groups could have been seen as a limitation given that the focus group method emphasises the importance of meeting in person [37], yet it was perceived as a strength as it enabled more participants to participate because it enabled attendance and saved time. Interaction in the groups was good, and participants were used to communicate via video conferencing systems due to the ongoing pandemic. Accordingly, the fact that the online focus groups worked well was also a strength. It could be viewed as a limitation that two of the participants had no experience in arranging group exercises before the pandemic. In retrospect, it would have been better to try to get two other participants who had experience both before and during the pandemic. Considering the other participants, it was not deemed relevant to exclude the focus groups in which these two took part. Instead, their impact on the study’s findings was controlled for afterwards.

## Conclusion

The findings of this study demonstrated strategies to foster empowerment and exercise habits among older persons and to strengthen the arrangement of group exercises over time. Person-centredness emerges as part of health-promoting strategies both at the individual and organisational level. Hence, the study contributes to an understanding of how person-centredness is significant when working with health-promotive interventions for healthy ageing. Accordingly, the study shows how stakeholders strive to facilitate social belonging and thus empowerment increased the possibilities for continued exercise habits. Though, there is a need of increased competence among stakeholders, to create more targeted and high-quality interventions for older persons. This in turn can contribute to promoting sustainable exercise habits among older persons and thereby also promote healthy ageing. This study also contributes to the understanding of how a strong supportive organisational system can lead to stability for the older persons as it enables them to continue exercising. Thus, an implication is to use a person-centred approach, both at individual and organisational level, when arranging health-promotive interventions, like group exercise, for older persons. However, more studies are needed to explore older persons’ own perspective regarding whether the group exercise activities via the meeting places promote health and are person-centred.

## Electronic supplementary material

Below is the link to the electronic supplementary material.

Supplementary Material 1

## Data Availability

The datasets analysed during the current study are available from the corresponding author on reasonable request.
