# Supplementary material for: Strategies for person-centredness at meeting places arranging group exercises for community-dwelling older persons– A focus group study from a stakeholder perspective
Source: BMC Public Health. 2025 Mar 13;25:998. doi: 10.1186/s12889-025-21843-z (PMC11908056; doi:10.1186/s12889-025-21843-z)
Supplement: Supplementary file 1 — Supplementary Material 1 [file 12889_2025_21843_MOESM1_ESM.docx]

# Additional file 1: Interview guide

## Introduction

All participants are asked to briefly present themselves and their role in arranging group exercises via the meeting places.

## Opening interview request

Please tell us about the group exercise activities that you are involved in via the municipal meeting places.

## Prerequisites

- How did the physical activities for the community-dwelling older persons via meeting places start in the municipality?
- What prerequisites do you think are required to arrange good physical activities for older persons?
- How do you work to ensure the quality of the physical activities?
- Have there been any obstacles to implementation that have led to the modification of the activities?

## Resources

- What resources are needed to implement physical activity? Premises, staff?
- Did you need involvement, support or resources from the community? If so, which?
- What happens in the case of absence, for example due to illness or when someone involved in the activity quits?

## Collaboration

- Are the group exercise activities arranged by the municipality or in collaboration with other stakeholders?
- Do you collaborate with other stakeholders? Which ones? How did such collaboration arise?
- What support is provided for arranging group exercises and how do you support the other stakeholders?

## Participation

- Who decided which activities to offer and on what basis?
- How do the older persons participate in the planning of the group exercises?
- How do the older persons get involved in the activities? Are they selected or can they sign up themselves?
- Can the older persons influence the arrangement of group exercises? E.g. time, place, organisation?

## Empowerment

- What feedback have you received on the activities from the older persons?
- How are the older persons affected by participating in the group exercises?
- Have you found that the older persons started thinking more about their health since they began participating in group exercises? Do you have any examples?

## Group exercises to promote health

- What is the overall objective of your organisation?
- What is health promotion, what does it mean to you?
- Is the effect of the physical training followed up by anyone? By whom and how?
- Do the activities continue over time? Can the older persons take part every term?
- How do you make it possible to invite new participants but at the same time give the older persons the opportunity to continue with the activity over time if they wish?
- What do you think is needed for the older persons to continue with the activities they have started?

## Closing interview question

- Is there anything important that you think we have omitted to discuss?

## Examples of prompting questions:

Can you give an example…?

What do you mean when you say…?

Can you tell me in which way…?
